# Impact of the Acceptance of Disability on Self-Esteem among Adults with Disabilities: A Four-Year Follow-Up Study

**DOI:** 10.3390/ijerph19073874

**Published:** 2022-03-24

**Authors:** Yun Hwa Jung, Soo Hyun Kang, Eun-Cheol Park, Suk-Yong Jang

**Affiliations:** 1 Department of Public Health, Graduate School, Yonsei University, Seoul 03722, Korea; yunhwa@yuhs.ac (Y.H.J.); kshyun@yuhs.ac (S.H.K.); 2 Institute of Health Services Research, Yonsei University, Seoul 03722, Korea; ecpark@yuhs.ac; 3 Department of Preventive Medicine, Yonsei University College of Medicine, Seoul 03722, Korea; 4 Department of Healthcare Management, Graduate School of Public Health, Yonsei University, Seoul 03722, Korea

**Keywords:** disability, adaptation, self-esteem, self-concept, disabled persons, adult

## Abstract

This study identified the acceptance of disability’s impact on self-esteem among adults with disabilities in South Korea. This is a four-year follow-up study that obtained data from the Panel Survey of Employment for Persons with Disabilities from 2017 to 2020. In total, 3329 individuals participated. Logistic regression examined the acceptance of disability’s effect on self-esteem. These variables were categorized based on the acceptance of disability (high→high, low→high, high→low, and low→low) and self-esteem (low and not low). Compared to the participants with a consistently high acceptance of disability, those with constantly low acceptance were 2.35 times (95% CI 1.81–3.04) more likely to have low self-esteem. When the acceptance of disability was low→high and high→low, the low self-esteem probability was 1.23 and 1.66 times, respectively. Low self-esteem was prominent for the following: men, 50–64-year olds, married, urban, economic activists, the mid–low household income category, and those with sensory disability. Acceptance of disability can adversely affect self-esteem when it is consistently low or changes from high to low. Among socio-economic factors, there were several risk factors that could make individuals more vulnerable to low self-esteem. Therefore, it is necessary to help people accept their disabilities to maintain healthy self-esteem levels.

## 1. Introduction

The prevalence of disability is 35.7% in Finland, 21.0% in the UK, 12.7% in the US, and 5.1% in Korea [1,2,3]. It is difficult to draw a simple comparison because the disability standards vary by country. Nevertheless, it is estimated that about 15.6% of the world’s population aged 15 and over have some disability [4]. In 2020, 87.9% of the disabilities in Korea were acquired, while 41.4% were caused by disease [5]. Additionally, an increase in life expectancy increases the risk of disability due to increased causes such as acquired diseases and accidents.

Disability is a controversial concept with various definitions; it is a complex medical and social problem. In recent years, it has shifted from a personal medical perspective to a structural and social one [4]. Restrictions resulting from persons with disabilities, including physical and mental impairments, are currently being considered by individuals and society as significant concerns. Despite changing definitions, it remains a fact that disability inevitably coexists with persons with disabilities, acquaintances, and their environment.

The acceptance of disability entails an acknowledgment of loss [6]. Accepting a disability requires individuals to avoid devaluing a person owing to their disability, hiding it due to shame, and overestimating the disability. It involves recognizing the inconvenience caused by the disability, attempting to recover, and accepting the reality and the restrictions accompanying it. Disability acceptance is essential for people with disabilities who experience social prejudice or frustration as it helps them recognize their worth and adapt to living in a society. According to previous studies, whether it is a congenital or an acquired disability, life’s direction changes depending on how one accepts the disability. The disability paradox theory refers to individuals who are satisfied with themselves, can attain their life goals, and enjoy a high quality of life despite or because of a disability [7]. Those who are more receptive to their disabilities have higher levels of self-esteem, social participation, and quality of life [8,9].

Self-esteem is a subjective attitude toward oneself, evaluated as acceptable or unacceptable regarding one’s own worth [10]. It develops over a long time, tends to remain stable, and is influenced by socio-economic factors [11,12]. High self-esteem contributes to improved performance, interpersonal relationships, happiness, and health [13]. In contrast, low self-esteem is associated with anxiety, depression, suicide, alcoholism, aggression, antisocial behavior, and delinquency [14,15,16]. Persons with disabilities should be especially wary of having low self-esteem because they are more vulnerable to psychological risks. Approximately 34.8% of people with disabilities experience social discrimination, and 61.5% consider themselves economically inferior. As they age, people with disabilities are more than twice as likely to be depressed or consider suicide than those without disabilities [17].

The acceptance of disability and low self-esteem, which are closely concerned with stigma, is self-perceived compared to others [18]. In previous studies, people with a history of mental illness tended to hide their illness to avoid stigma-induced insults, rejection, devaluation, and discrimination [19,20]. Children with learning disabilities feel ashamed of their academic performance [21]. Nevertheless, they actively hid their disabilities from their classmates and teachers, since labeling a learning disability could lead to social stigmas such as stupidity, carelessness, and laziness [22]. In other words, whether people with disabilities can hide their disability can affect their acceptance of disability and self-esteem. We designed this study to adjust for disability type.

Few studies examined the association between the acceptance of disability and self-esteem based on specific disabilities and gender [23,24,25]. Furthermore, macroscopic research of disability existence and self-esteem are relatively scarce. In addition, the analysis of each item of the acceptance of disability scale or the self-esteem scale was inadequately conducted thus far. Therefore, we investigated the impact of acceptance of disability on self-esteem among adults with disabilities from a macro perspective. Additionally, we analyzed all items of the acceptance of disability and self-esteem scales. We hypothesized that low acceptance of disability would cause low self-esteem among adults with disabilities.

We analyzed low self-esteem according to changes in acceptance of disability as the main analysis. Changes in acceptance of disability were categorized into four categories; “High→High”, “Low→High”, “High→Low”, and “Low→Low”. For example, the “High→Low” status means that acceptance of disability was high in the past but low in the current. Low self-esteem was categorized as “yes” and “no”. In the main analysis, acceptance of disability and low self-esteem were distinguished by calculating the sum of the acceptance of disability scale and the Rosenberg self-esteem scale, which consisted of the Likert scale. As a further analysis, we analyzed each item of those scales to identify the factors that a particular impact had.

## 2. Materials and Methods

### 2.1. Data

The research data for this study were obtained from the second wave of the Panel Survey of Employment for Persons with Disabilities (PSED) spanning over 4 years in Korea. The data from 2017, when the variable of interest (acceptance of disability) was able to obtain this study, to 2020, the latest data, were used. The PSED is a representative panel survey for individuals with disabilities in Korea conducted by the Korea Employment Agency for Persons with Disabilities (KEAD: Seongnam, Korea) and includes repeatedly measured personal longitudinal data [26]. The data provides basic statistics regarding the economic activity of people with disabilities and personal and environmental information. The survey method employed was a direct one-on-one interview conducted using Tablet PC-Assisted Personal Interviewing (TAPI). The researchers could input the interviewees’ responses through the TAPI program and logically minimize errors. At the time of the COVID-19 pandemic in 2020, online and telephonic surveys were conducted separately for interviewees who refused face-to-face assessments. This study did not require approval or prior consent from the Institutional Review Board. The PSED was a secondary dataset available in the public domain and de-identified all participants, ensuring their data’s anonymity and confidentiality.

### 2.2. Participants

In 2017, the baseline for this study, 4214 adults, were extracted from the Ministry of Health and Welfare’s list of registered persons with disabilities. In 2020, the effective sample retention rate was 87.2%, excluding 885 participants who died or cancelled their disability registration, thus explaining the missing data. Overall, 3329 participants were analyzed in 2017 (2160 men and 1169 women).

### 2.3. Variables

The acceptance of disability was assessed using the 12-item Acceptance of Disability Scale [27,28], with responses rated on a 5-point Likert scale. As a result of the receiver operating characteristic analysis based on all its items, the area under the curve was 0.931, sensitivity 0.699, and specificity 1.000 in 36.999 cut-off points. The authors selected 37 points as the cut-off score to distinguish the acceptance of disability degree: low = 12–36 points and high = 37–60 points. Self-esteem was the dependent variable assessed using the 10-item Rosenberg Self-esteem Scale, with responses rated on a 4-point Likert scale. Self-esteem was divided into low self-esteem (0–14 points) and not low (15–30 points) [29].

The covariates were demographic (gender and age) and socioeconomic characteristics (region, marital status, household income, economic activity, and educational level), mental health (perceived stress), and disability variables (the disability’s type, period, and severity). The Korean Ministry of Health and Welfare categorized the disability types as follows: internal (epilepsy, fistula of the intestine and urinary system, and kidney, cardiac, respiratory, and hepatic dysfunctions), external (physical and brain lesion disability), sensory (visual, hearing, and language impairments), and mental (mental disability (retardation) and autism).

### 2.4. Statistical Analysis

Chi-square tests were conducted to analyze the participants’ baseline characteristics according to the study variables. A generalized estimating equation model was applied to evaluate the acceptance of disability’s impact on self-esteem. Subgroup analysis stratified the participants by gender, economic status, and the disability’s type and severity. Moreover, it was also performed for each item of the Acceptance of Disability Scale and the Rosenberg Self-esteem Scale. The results included adjusted odds ratios (AORs) and 95% confidence intervals (95% CIs). Further, statistical significance was set at *p* ≤ 0.05. All statistical analyses were performed using Statistical Analysis Software (SAS), version 9.4 (SAS Institute Inc., Cary, NC, USA).

## 3. Results

Among the 3329 participants included in this study, 2160 were men (64.9%) and 1169 were women (35.1%); their mean ages were 46.0 ± 11.9 and 49.0 ± 12.9 years, respectively. Regarding the acceptance of disability, the proportion of low self-esteem increased in the order of high→high (46.1%), low→high (17.8%), high→low (14.1%), and low→low (22.1%), while their section’s gender ratio was similar to the overall gender ratio (Table 1).

Those who demonstrated low disability acceptance tended to have lower self-esteem compared to those who did not. When the acceptance of disability was low→high, the AOR was 1.23 (95% CI 0.92–1.64), referring to being consistently high. The subsequent prominent AOR was 1.66 (95% CI 1.33–2.08) in high→low. Consistently low acceptance of disability was the most pronounced (AOR 2.35, 95% CI 1.81–3.04). Participants were more likely to have low acceptance of disability than high acceptance of disability (high→high: 8.5%, low→high: 17.1%, high→low: 26.9%, low→low: 43.7%) (Table 2, Supplementary Table S1).

In the sub-analysis of the covariates according to the acceptance of disability, some variables for which the socioeconomic status (SES) was considered superior were vulnerable to low self-esteem. Being male, married, urban residents, and economic activists tended to be risks for low self-esteem in cases with low acceptance of disability than consistently high acceptance of disability. The trend increased in the order of low→high, high→low, with a consistently low acceptance of disability. It was prominent in consistently low acceptance of disability (men: AOR 2.55, 95% CI 1.87–3.48; married individuals: AOR 3.01, 95% CI 2.14–4.24; urban residents: AOR 2.97, 95% CI 2.07–4.27; economic activists: AOR 2.76, 95% CI 1.93–3.94). This tendency of low self-esteem as exposure to less acceptance of disability was also observed in 50–64-year olds (low→low: AOR 3.30, 95% CI 2.37–4.58), those with a sensory disability (low→low: AOR 3.63, 95% CI 2.32–5.68), and those having mid–low household income (low→low: AOR 3.93, 95% CI 2.64–5.84) (Table 3, Supplementary Table S2).

In the first additional analysis, low self-esteem was analyzed for each item of the Acceptance of Disability Scale. The risk of low self-esteem was prominent in the item “I cannot get along well with people because of my disability” (AOR 8.88, 95% CI 5.82–13.53) (Table 4).

In the second additional analysis, each item of Rosenberg’s Self-Esteem Scale was analyzed according to acceptance of disability. The more exposed one was to low acceptance of disability, the more prominent the risk of not experiencing self-satisfaction (low→high: AOR 1.54, 95% CI 1.25–1.90; high→low: AOR 3.44, 95% CI 2.83–4.18; low→low: AOR 6.19, 95% CI 5.07–7.56) (Table 5).

## 4. Discussion

Low self-esteem was affected in the following order: consistently high, from low to high, from high to low, and consistently low acceptance of disability. The impact was evident in the following cases: men, 50–64-year olds, married individuals, urban residents, economic activists, and those with mid–low household income and a sensory disability. The people with disabilities who disagreed that honesty is more important than a disability, in terms of acceptance of disability, were at a higher risk of having low self-esteem; however, those who were dissatisfied with themselves, in terms of self-esteem, tended to have a lower acceptance of disability.

In this study, 54.1% of the participants had reduced disability acceptance currently or in the past year, and 67.9% had low self-esteem. This is consistent with the findings of previous studies reporting that individuals with disabilities had lower self-esteem than those without disabilities [24,25,30]. It was also in line with the hypothesis that a lower acceptance of disability causes lower self-esteem.

Acceptance of disability and self-esteem had a dose–response relationship. The likelihood of low self-esteem increased as the acceptance of disability changed from low to high, from high to low, and consistently low when acceptance of disability was consistently high. The distribution of disability acceptance was more prevalent in the low than in the high self-esteem individuals. This is one of the reasons why the acceptance of disability is important to people with disabilities for their mental health. In addition, low self-esteem was more prominent when the status of acceptance of disability went from high to low than from low to high. It can be inferred that current disability acceptance contributes more to present self-esteem than in the past.

Despite the limitations of various disabilities, people with disabilities experience success in interpersonal relationships, achievements, and overcoming distress. The accumulation of positive experiences contributes to the acceptance of their disability, which in turn leads to the development of self-esteem. Since self-esteem is an “energy store” filled with effective self-satisfaction [31], the acceptance of disability caused by the collection of successful experiences can lead to high self-esteem. An increase in acceptance of disability adds positive energy into the self-esteem account, whereas a decrease causes a deficit in the account. This may reduce the resistance to negative internal or external factors.

The factors that particularly influenced low self-esteem following disability acceptance comprised men, 50–64-year olds, married individuals, urban residents, economic activists, and those with mid–low household income and a sensory disability. In psychiatry, men, married people, urban residents, and economically active persons are often considered to have more favorable SES [32,33,34,35].

Nevertheless, it can be considered that disabilities in 9 out of 10 people with disabilities are acquired, and 62.5% of them experience the onset of the disability between the ages of 20–59 years. A person with a high SES may experience a higher sense of loss and deprivation by becoming socially vulnerable after the sudden appearance of a disability (acquired disability causes: accident 37%, disease 36.4%) [5]. Therefore, it may require more transitional time and effort for the acceptance of disability or healthy self-esteem.

A society of “normality” centered on people without disabilities isolates those with disabilities and makes them feel alienated. In the analysis of social discrimination by disability type, the most social discrimination was experienced regarding sensory impairments [3]. The order in which the disability types were socially discriminated against was the same as that in which they were at risk for low self-esteem when the acceptance of disability declined (Sensory > External > Internal > Mental). Concerning sensory disabilities, including visual and hearing impairments, they are difficult to hide at the communication stage. External disabilities make it easier for others to visually recognize their disability. Conversely, internal or mental disabilities are relatively less exposed. In other words, persons with disabilities may have more vulnerable self-esteem concerning the acceptance of the disability when their disabilities are prone to exposure to others.

Lifetime wealth may be the reason why the disability acceptance and self-esteem of 50–64-year olds and mid–low income households are impacted. According to the life cycle hypothesis, the aforementioned age group is considered the last period in the wealth cycle, when income outweighs consumption due to retirement [36]. Moreover, the average additional cost of a disability annually is about KRW 1.98 million (USD 1.76 million), specifically aimed at medical care (58.1% of medical expenses) [3]. Low–middle income groups, where social insurance coverage is likely to be a blind spot, may aggravate anxiety and increase the desire to avoid spending without new income. Anxiety and stress caused by a disability that prevents a person from doing what they want using their own efforts can lead to reality avoidance, self-denial, or aversion. This can become an obstacle to acknowledging a disability; although it does not physically exaggerate it; however, it can be psychologically demeaning, thus leading to low self-esteem.

On the Acceptance of Disability Scale, adults who struggled because of their disabilities were more likely to have low self-esteem. This may be because they are more clearly aware of the alienation in the process of hiding the disabilities’ limitations. As a means of overcoming the feelings of inferiority, they refuse to accept their disability by distorting the reality or deluding themselves, thus causing low self-esteem. Nonetheless, it may be because disability leads to social discrimination, abuse, stigma, and ill-treatment, resulting in isolation and hostility. Economic exploitation was ranked first in the type of disability abuse [37]. It refers to unreasonable treatment without a reasonable cause, including extortion of property, transfer of debts, refusal to hire or promote, and low wages and services. Regarding the social stigma that neglects the disabled, they may experience society’s absurdity and psychological distance.

In the additional analysis of self-esteem factors, the people with disabilities who did not experience self-satisfaction tended to have a reduced acceptance of their disability. Self-deprecation, self-hatred, shame, and self-guilt associated with poor self-satisfaction are factors to be aware of in recognition of a disability. This is because they may be triggers of depression and suicide while also causing low self-esteem. Discrimination by mainstream groups internalizes antipathy toward persons with disabilities or racial, gender, and religious groups [38]. Self-antagonism is particularly high when dissatisfied with hard-to-change factors, such as disability, height, or gender identity. Therefore, individuals with negative self-awareness may be unable to objectively accept their disability and are at a high risk of having low self-esteem. In addition, they cannot afford to be tormented internally (emotional turmoil); therefore, they may lash out by expressing selfish behaviors and aggression externally. Thus, it is also worth paying attention to the improvement of low self-esteem following the acceptance of disability.

This study has some limitations that should be discussed. First, although various efforts were made by the KEAD to minimize bias (e.g., by educating the survey investigators), they could not exclude response or recall biases that might have influenced the results. Second, this research analyzed the diverse types of disability by grouping them into four categories for examining the number of participants in each type. It is necessary to explore how self-esteem affects the acceptance of disability among the specific types. Focusing on them might reveal differences according to the specific types of disability. Therefore, caution should be exercised when interpreting the findings.

## 5. Conclusions

Low acceptance of disability contributed to low self-esteem. Men, 50–64-year olds, married individuals, urban residents, economic activists, and those with mid–low household income and a sensory disability were vulnerable to low self-esteem based on their acceptance of disability. As some factors are considered superior SES, they may weaken the self-esteem of people with disabilities; hence, it is necessary to support their mental health so that there are no blind spots. Those individuals with disabilities who could not get along with others were at a higher risk of having low self-esteem. They may feel alienated and hide their disability or may experience isolation and hostility due to social absurdity. Regarding self-esteem, those with disabilities without self-satisfaction had reduced acceptance of disability; further, when self-denial was maximized, there was a pathological risk of depression and suicide. This may also be accompanied by the social risks of selfish behaviors and aggression. Therefore, it is necessary to care for people with disabilities to ensure they have high self-esteem by accepting their disabilities in a healthy way, not only at the individual level but also at the societal level.

## Figures and Tables

**Table 1:** Baseline characteristics of the study population (baseline, 2017).

Variables	Acceptance of Disability (2016→2017)										
	Total		High→High		Low→High		High→Low		Low→Low		
	N	%	N	%	N	%	N	%	N	%	*p*-Value
**Total**	3329	100.0	1534	46.1	592	17.8	468	14.1	735	22.1	
**Gender**											0.3211
Men	2160	64.9	1014	66.1	388	65.5	289	61.8	469	63.8	
Women	1169	35.1	520	33.9	204	34.5	179	38.2	266	36.2	
**Age**											0.0952
19–29	133	4.0	52	3.4	28	4.7	23	4.9	30	4.1	
30–49	1104	33.2	475	31.0	205	34.6	167	35.7	257	35.0	
50–64	1697	51.0	830	54.1	292	49.3	225	48.1	350	47.6	
65–	395	11.9	177	11.5	67	11.3	53	11.3	98	13.3	
**Marital status**											<0.0001
Married	1569	47.1	906	59.1	267	45.1	182	38.9	214	29.1	
Single, widow, divorced, separated	1760	52.9	628	40.9	325	54.9	286	61.1	521	70.9	
**Region**											0.0020
Urban area	1618	48.6	703	45.8	290	49.0	225	48.1	400	54.4	
Rural area	1711	51.4	831	54.2	302	51.0	243	51.9	335	45.6	
**Educational level**											<0.0001
University or above	1066	32.0	660	43.0	147	24.8	134	28.6	125	17.0	
High school or below	2263	68.0	874	57.0	445	75.2	334	71.4	610	83.0	
**Economic activity**											<0.0001
Active	1735	52.1	1068	69.6	275	46.5	194	41.5	198	26.9	
Non-active	1594	47.9	466	30.4	317	53.5	274	58.5	537	73.1	
**Household income**											<0.0001
High	768	23.1	498	32.5	105	17.7	81	17.3	84	11.4	
Mid–high	889	26.7	476	31.0	158	26.7	124	26.5	131	17.8	
Mid–low	833	25.0	337	22.0	167	28.2	122	26.1	207	28.2	
Low	839	25.2	223	14.5	162	27.4	141	30.1	313	42.6	
**Stress**											0.0003
Less	342	10.3	184	12.0	71	12.0	32	6.8	55	7.5	
Much	2987	89.7	1350	88.0	521	88.0	436	93.2	680	92.5	
**Disability type**											<0.0001
Internal	275	8.3	109	7.1	44	7.4	42	9.0	80	10.9	
External	1962	58.9	1007	65.6	332	56.1	278	59.4	345	46.9	
Sensory	720	21.6	340	22.2	142	24.0	92	19.7	146	19.9	
Mental	372	11.2	78	5.1	74	12.5	56	12.0	164	22.3	
**Disability period**											0.0018
Congenital	327	9.8	134	8.7	46	7.8	55	11.8	92	12.5	
≤5 years	230	6.9	96	6.3	41	6.9	33	7.1	60	8.2	
5–10 years	511	15.3	260	16.9	93	15.7	76	16.2	82	11.2	
>10 years	2261	67.9	1044	68.1	412	69.6	304	65.0	501	68.2	
**Disability severity**											<0.0001
Low	2105	63.2	1146	74.7	357	60.3	283	60.5	319	43.4	
High	1224	36.8	388	25.3	235	39.7	185	39.5	416	56.6	

**Table 2:** Generalized linear model using the GEE with low self-esteem in 2017–2020.

Variables	Low Self-Esteem			
	N ^a^	% ^b^	Adjusted OR (95% CI)	
**Acceptance of disability (2016→2017)**				
High→High	130	8.5	1.00	
Low→High	101	17.1	1.23	(0.92–1.64)
High→Low	126	26.9	1.66	(1.33–2.08)
Low→Low	321	43.7	2.35	(1.81–3.04)

^a^ The number of respondents who had low self-esteem at the results of the self-esteem scale in the baseline year (2017). ^b^ In the column, the percentage of the low self-esteem on the self-esteem scale.

**Table 3:** Subgroup analysis using the GEE of low self-esteem according to acceptance of disability change in 2017–2020 ^a^.

Variables	Low Self-Esteem						
	Acceptance of Disability						
	High→High	Low→High		High→Low		Low→Low	
	Adjusted OR (95% CI)						
**Gender**							
Men	1.00	1.36	(0.95–1.93)	1.84	(1.38–2.46)	2.55	(1.87–3.48)
Women	1.00	1.06	(0.64–1.75)	1.38	(0.98–1.94)	2.09	(1.34–3.27)
**Age**							
19–29	1.00	1.12	(0.49–2.53)	1.77	(0.95–3.26)	2.54	(1.34–4.80)
30–49	1.00	1.03	(0.66–1.60)	1.41	(1.02–1.95)	2.05	(1.39–3.01)
50–64	1.00	1.59	(1.02–2.47)	2.25	(1.61–3.14)	3.30	(2.37–4.58)
65–	1.00	1.59	(0.75–3.40)	1.58	(0.79–3.17)	1.98	(0.95–4.12)
**Marital status**							
Married	1.00	1.23	(0.79–1.92)	1.67	(1.19–2.35)	3.01	(2.14–4.24)
Single, widow, divorced	1.00	1.13	(0.77–1.66)	1.59	(1.18–2.13)	1.98	(1.39–2.83)
**Region**							
Urban area	1.00	1.46	(1.00–2.11)	1.82	(1.36–2.42)	2.97	(2.07–4.27)
Rural area	1.00	1.04	(0.69–1.59)	1.59	(1.16–2.18)	2.01	(1.42–2.86)
**Economic activity**							
Active	1.00	1.59	(1.02–2.46)	2.22	(1.52–3.24)	2.76	(1.93–3.94)
Non-active	1.00	1.04	(0.73–1.48)	1.41	(1.08–1.84)	2.10	(1.52–2.89)
**Household income**							
High	1.00	1.21	(0.64–2.31)	2.62	(1.52–4.52)	2.91	(1.64–5.15)
Mid–high	1.00	0.92	(0.54–1.54)	1.76	(1.11–2.81)	2.78	(1.73–4.46)
Mid–low	1.00	1.68	(1.06–2.68)	2.18	(1.45–3.27)	3.93	(2.64–5.84)
Low	1.00	0.98	(0.62–1.56)	1.20	(0.84–1.71)	1.53	(1.01–2.32)
**Disability type**							
Internal	1.00	0.75	(0.30–1.85)	1.77	(0.91–3.46)	3.24	(1.75–5.97)
External	1.00	1.21	(0.81–1.81)	1.67	(1.22–2.29)	2.25	(1.59–3.20)
Sensory	1.00	1.56	(1.02–2.37)	1.87	(1.22–2.88)	3.63	(2.32–5.68)
Mental	1.00	1.06	-	1.43	-	1.75	-

^a^ Adjusted for demographic, socioeconomic, health-related factors, and housing-related factors as potential confounders.

**Table 4:** Contents of self-esteem degree and variable of interest analysis concerning the acceptance of disability scale factors ^a,b,c^.

Variables	Self-Esteem		
	Not Low	Low	
	Adjusted OR (95% CI)		
**Acceptance of disability questions (answering disagree)**			
I cannot get along well with people because of my disability. (R)	1.00	8.88	(5.82–13.53)
My disability made me think of the world more broadly.	1.00	1.39	(0.83–2.32)
I feel bad when I can not do something because of my disability. (R)	1.00	2.06	(1.54–2.77)
I do not suffer because of my disability.	1.00	2.34	(1.67–3.27)
I am a person with a disability, but I am satisfied with my life.	1.00	3.14	(2.02–4.90)
How I live my life is more important to me than the disability itself.	1.00	2.29	(1.31–3.98)
Disability has the greatest impact on my life. (R)	1.00	2.08	(1.56–2.78)
Honesty is more important than the disability itself.	1.00	1.90	(1.15–3.13)
There are many things in life that are far more important than appearance.	1.00	1.41	(0.86–2.31)
There are so many fun things that I forget that I am disabled and live.	1.00	2.99	(2.19–4.08)
Though I have a disability, my life is full.	1.00	3.55	(2.59–4.86)
I am uncomfortable because of my disability, but I can do anything if I put my mind to it.	1.00	3.51	(2.52–4.89)

^a^ Items marked with (R) are reverse-coded. ^b^ Adjusted for demographic, socioeconomic, health-related factors, and housing-related factors as potential confounders. ^c^ When the acceptance of disability was low in the analysis of each item of the acceptance of disability scale, the self-esteem status was indicated.

**Table 5:** Contents of dependent variable analysis concerning self-esteem scale factors according to acceptance of disability ^a,b,c^.

Variables	Acceptance of Disability						
	High→High	Low→High		High→Low		Low→Low	
	Adjusted OR (95% CI)						
**Self-esteem questions (answering disagree)**							
I am a person of worth.	1.00	1.64	(1.18–2.28)	3.10	(2.38–4.04)	3.37	(2.55–4.45)
I have a number of good qualities.	1.00	1.73	(1.34–2.24)	2.25	(1.76–2.87)	2.69	(2.12–3.40)
I am inclined to feel that I am a failure. (R)	1.00	1.14	(0.94–1.38)	1.19	(0.99–1.43)	1.23	(1.02–1.47)
I am able to do things as well as most other people.	1.00	1.60	(1.30–1.97)	2.78	(2.30–3.36)	4.52	(3.73–5.47)
I feel I do not have much to be proud of. (R)	1.00	1.18	(1.00–1.39)	1.23	(1.03–1.47)	1.68	(1.40–2.02)
I have a positive attitude toward myself.	1.00	1.68	(1.30–2.16)	2.95	(2.39–3.64)	4.15	(3.29–5.25)
I am satisfied with myself.	1.00	1.54	(1.25–1.90)	3.44	(2.83–4.18)	6.19	(5.07–7.56)
I wish I could have more respect for myself. (R)	1.00	0.97	(0.79–1.19)	0.55	(0.45–0.67)	0.58	(0.47–0.70)
I certainly feel useless at times. (R)	1.00	1.14	(0.95–1.38)	1.27	(1.07–1.50)	1.29	(1.07–1.56)
At times I think I am no good at all. (R)	1.00	1.13	(0.94–1.37)	1.01	(0.85–1.22)	0.69	(0.57–0.84)

^a^ Items marked with (R) are reverse-coded. ^b^ Adjusted for demographic, socioeconomic, health-related factors, and housing-related factors as potential confounders. ^c^ When the acceptance of disability was changed by each item of the self-esteem scale, the self-esteem status was indicated.

## Data Availability

Publicly available datasets were analyzed in this study. This data can be found here: [https://edi.kead.or.kr].

## Supplementary Materials

The following supporting information can be downloaded at: https://www.mdpi.com/article/10.3390/ijerph19073874/s1, Supplementary Table S1. Generalized linear model using the GEE with low self-esteem in 2017–2020; Supplementary Table S2. Subgroup analysis using the GEE of low self-esteem according to acceptance of disability change in 2017–2020.
