# Supplementary material for: Impact of the Acceptance of Disability on Self-Esteem among Adults with Disabilities: A Four-Year Follow-Up Study
Source: Int J Environ Res Public Health. 2022 Mar 24;19(7):3874. doi: 10.3390/ijerph19073874 (PMC8997373; doi:10.3390/ijerph19073874)
Supplement: Supplementary file 1 [file ijerph-19-03874-s001.zip › ijerph-1611888-supplementary.pdf]

Supplementary Table S1. Generalized linear model using the GEE with low self-esteem in 2017–2020.

Variables	Low self-esteem				
	N[a]	%[b]	Adjusted OR (95% CI)		
**Acceptance of disability (2016→2017)**					
High→High	130	8.5	1.00		
Low→High	101	17.1	1.23	(0.92 –	1.64)
High→Low	126	26.9	1.66	(1.33 –	2.08)
Low→Low	321	43.7	2.35	(1.81 –	3.04)
**Gender**					
Men	442	20.5	1.00		
Women	236	20.2	0.83	(0.68 –	1.01)
**Age**					
19–29	31	23.3	1.09	(0.81 –	1.45)
30–49	232	21.0	0.99	(0.73 –	1.34)
50–64	328	19.3	0.72	(0.46 –	1.13)
65–	87	22.0	1.00		
**Marital status**					
Married	177	11.3	1.00		
Single, widow, divorced, separated	501	28.5	1.27	(1.03 –	1.57)
**Region**					
Urban area	364	22.5	1.00		
Rural area	314	18.4	0.81	(0.67 –	0.97)
**Educational level**					
University or above	144	13.5	1.00		
High school or below	534	23.6	1.19	(0.94 –	1.52)
**Economic activity**					
Active	149	8.6	1.00		
Non-active	529	33.2	2.63	(2.14 –	3.24)
**Household income**					
High	73	9.5	1.00		
Mid-high	122	13.7	1.34	(1.01 –	1.77)
Mid-low	195	23.4	1.55	(1.20 –	2.02)
Low	288	34.3	1.85	(1.38 –	2.47)
**Stress**					
Less	52	15.2	1.00		
Much	626	21.0	1.47	(1.11 –	1.96)
**Disability type**					
Internal	121	16.8	1.00		
External	323	16.5	1.24	(0.90 –	1.71)
Sensory	162	43.5	1.16	(0.82 –	1.64)
Mental	72	26.2	2.19	(1.53 –	3.13)
**Disability period**					
Congenital	84	25.7	1.00		
≤5 years	453	20.0	1.37	(0.76 –	2.46)
5–10 years	86	16.8	1.01	(0.68 –	1.49)
> 10 years	55	23.9	1.13	(0.83 –	1.54)
**Disability severity**					
Low	314	14.9	1.00		
High	364	29.7	1.24	(0.97 –	1.58)

[a] The number of respondents who had the low self-esteem at the results of the self-esteem scale in baseline year (2017). [b] In the column, the percentage of the low self-esteem on the self-esteem scale.

Supplementary Table S2. Subgroup analysis using the GEE of low self-esteem according to acceptance of disability change in 2017-2020[a].

Variables	Low self-esteem						
	Acceptance of disability						
	High→High	Low→High		High→Low		Low→Low	
	Adjusted OR (95% CI)						
**Gender**							
Men	1.00	1.36	(0.95 – 1.93)	1.84	(1.38 – 2.46)	2.55	(1.87 – 3.48)
Women	1.00	1.06	(0.64 – 1.75)	1.38	(0.98 – 1.94)	2.09	(1.34 – 3.27)
**Age**							
19–29	1.00	1.12	(0.49 – 2.53)	1.77	(0.95 – 3.26)	2.54	(1.34 – 4.80)
30–49	1.00	1.03	(0.66 – 1.60)	1.41	(1.02 – 1.95)	2.05	(1.39 – 3.01)
50–64	1.00	1.59	(1.02 – 2.47)	2.25	(1.61 – 3.14)	3.30	(2.37 – 4.58)
65–	1.00	1.59	(0.75 – 3.40)	1.58	(0.79 – 3.17)	1.98	(0.95 – 4.12)
**Marital status**							
Married	1.00	1.23	(0.79 – 1.92)	1.67	(1.19 – 2.35)	3.01	(2.14 – 4.24)
Single, widow, divorced	1.00	1.13	(0.77 – 1.66)	1.59	(1.18 – 2.13)	1.98	(1.39 – 2.83)
**Region**							
Urban area	1.00	1.46	(1.00 – 2.11)	1.82	(1.36 – 2.42)	2.97	(2.07 – 4.27)
Rural area	1.00	1.04	(0.69 – 1.59)	1.59	(1.16 – 2.18)	2.01	(1.42 – 2.86)
**Educational level**							
University or above	1.00	1.61	(0.90 – 2.89)	1.94	(1.20 – 3.15)	2.79	(1.69 – 4.60)
High school or below	1.00	1.14	(0.83 – 1.59)	1.63	(1.27 – 2.09)	2.27	(1.70 – 3.03)
**Economic activity**							
Active	1.00	1.59	(1.02 – 2.46)	2.22	(1.52 – 3.24)	2.76	(1.93 – 3.94)
Non-active	1.00	1.04	(0.73 – 1.48)	1.41	(1.08 – 1.84)	2.10	(1.52 – 2.89)
**Household income**							
High	1.00	1.21	(0.64 – 2.31)	2.62	(1.52 – 4.52)	2.91	(1.64 – 5.15)
Mid-high	1.00	0.92	(0.54 – 1.54)	1.76	(1.11 – 2.81)	2.78	(1.73 – 4.46)
Mid-low	1.00	1.68	(1.06 – 2.68)	2.18	(1.45 – 3.27)	3.93	(2.64 – 5.84)
Low	1.00	0.98	(0.62 – 1.56)	1.20	(0.84 – 1.71)	1.53	(1.01 – 2.32)
**Stress**							
Less	1.00	1.60	(0.71 – 3.60)	2.73	(1.20 – 6.20)	2.07	(0.87 – 4.94)
Much	1.00	1.18	(0.88 – 1.58)	1.61	(1.27 – 2.04)	2.42	(1.88 – 3.10)

**Disability type**													
Internal	1.00	0.75	(0.30	–	1.85)	1.77	(0.91	–	3.46)	3.24	(1.75	–	5.97)
External	1.00	1.21	(0.81	–	1.81)	1.67	(1.22	–	2.29)	2.25	(1.59	–	3.20)
Sensory	1.00	1.56	(1.02	–	2.37)	1.87	(1.22	–	2.88)	3.63	(2.32	–	5.68)
Mental	1.00	1.06	–	–	–	1.43	–	–	–	1.75	–	–	–
**Disability period**													
Congenital	1.00	1.41	(0.65	–	3.06)	1.79	(0.82	–	3.90)	2.99	(1.64	–	5.42)
≤5 years	1.00	0.02	(0.00	–	0.23)	1.58	(0.61	–	4.10)	5.75	(2.26	–	14.62)
5–10 years	1.00	0.84	(0.40	–	1.75)	1.15	(0.71	–	1.86)	0.91	(0.34	–	2.42)
> 10 years	1.00	1.35	(0.97	–	1.87)	1.82	(1.40	–	2.36)	2.61	(1.97	–	3.46)
**Disability severity**													
Low	1.00	1.34	–	–	–	1.88	–	–	–	2.85	–	–	–
High	1.00	1.09	(0.71	–	1.68)	1.46	(1.08	–	1.98)	1.88	(1.28	–	2.75)

[a] Adjusted for demographic, socioeconomic, health-related factors, and housing-related factors as potential confounders.
